# Female bias in an immigratory population of *Cnaphalocrocis medinalis* moths based on field surveys and laboratory tests

**DOI:** 10.1038/s41598-019-54721-x

**Published:** 2019-12-05

**Authors:** Jia-Wen Guo, Fan Yang, Ping Li, Xiang-Dong Liu, Qiu-Lin Wu, Gao Hu, Bao-Ping Zhai

**Affiliations:** 1 Key Laboratory of Integrated Management of Crop Diseases and Pests (Ministry of Education), College of Plant Protection, Nanjing Agricultural University, Nanjing, China; 2 Vegetable Research Institute, Wuhan Academy of Agricultural Science and Technology, Wuhan, China; 3 State Key Laboratory for Biology of Plant Diseases and Insect Pests, Institute of Plant Protection, Chinese Academy of Agricultural Sciences, Beijing, China

**Keywords:** Animal migration, Behavioural ecology, Entomology

## Abstract

Sex ratio bias is common in migratory animals and can affect population structure and reproductive strategies, thereby altering population development. However, little is known about the underlying mechanisms that lead to sex ratio bias in migratory insect populations. In this study, we used *Cnaphalocrocis medinalis*, a typical migratory pest of rice, to explore this phenomenon. A total of 1,170 moths were collected from searchlight traps during immigration periods in 2015–2018. Females were much more abundant than males each year (total females: total males = 722:448). Sex-based differences in emergence time, take-off behaviour, flight capability and energy reserves were evaluated in a laboratory population. Females emerged 0.78 days earlier than males. In addition, the emigratory propensity and flight capability of female moths were greater than those of male moths, and female moths had more energy reserves than did male moths. These results indicate that female moths migrate earlier and can fly farther than male moths, resulting more female moths in the studied immigratory population.

## Introduction

To ensure the survival, reproduction and growth of their populations, animals have evolved a series of adaptive life history strategies, such as sex differences in migratory strategies. Sex ratio bias is common in migratory animals and affects population development^1–3^. sex differences in reproductive behaviour and life history lead animals to adjust the sex structure during migration to change population structure. These changes affect individual fertility and population growth rates, which in turn influence population persistence^3–5^. Long-term monitoring studies have observed sex ratio bias in a number of migratory insect species. The sex ratios of light-trapped insects have been found to differ among species^6,7^. Some studies have found that males are captured by light traps more frequently than are females in some nocturnal insect species^8^; examples include *Parnassius mnemosyne*^9^, *Scotia segetum*^6^ and *Spodoptera exigua*^10^. However, female-biased migratory insect populations also exist, such as populations of *Aphid parasites*^11^, *Aphidus avenae*^12^, *Sogatella furcifera* and *Nilaparvata lugens*^13^. However, most previous studies analysed the phenomenon of sex ratio bias for the entire migration process, and little research has focused on this phenomenon during the immigratory period.

Sex differences in migration strategies may result in different times of arrival to the breeding area between males and females, which would affect the sex ratio of the animals during the immigration period. Females are responsible for most of the gene flow and dispersal in migratory insects (Johnson 1963), and the number of females often affects population size, which influences population growth. In some migratory animals, females reach the breeding ground earlier than males. Female bias in immigration has been reported in birds, including *Aegolius acadicus*^14^, *Actitis macularia*^15^ and *Phalaropus lobatus*^16^, and in *Helicoverpa armigera*, a facultatively migratory insect^17^. However, it remains unknown whether female bias occurs among obligate migrants of immigratory populations. If there is female bias in obligately migratory insects, what is the cause? We speculate that there may be several reasons to expect female bias in obligately migratory insects: (i) Females might emerge earlier than males. In many insects, females and males do not all appear at the same time in the same season, and the male and female emergence times are often not synchronized^18^. For example, in *H. armigera*, females tend to emerge earlier than males^19^, as observed in other, non-migratory insects, such as *Drosophila melanogaster*^20^ and *Oiketicus kirbyi*^21^. In migratory insects, females may emerge earlier than males. (ii) The emigratory propensity of females might be greater than that of males. Many studies have shown that females are more likely to disperse than males because the former need to fly for long periods to locate suitable oviposition sites^22–25^. Although close-range dispersal behaviour differs from long-distance migration, females of migratory insects may be more inclined to emigrate than males. (iii) The flight capability of females might be greater than that of males. This phenomenon is common in some non-migrating insects, such as *Eretmocerus eremicus*^26^ and *Bactrocera dorsalis*^27^. Females might fly farther than males. (iv) Females might have greater energy reserves than males, allowing them to fly farther. Flight potential is an important outcome of the state of reserves^28^. Because of their need to invest considerable energy into migration, migrants tend to consume more energy than residents^29,30^. The amount of energy resources used differs between the sexes, with some females consuming more energy resources than males^31^. In migratory insects, females may need to store more energy than males to allow long-range migration.

Here, the rice leaf folder, *Cnaphalocrocis medinalis* (Lepidoptera: Pyralidae), an obligate migrant, was studied to explore sex ratio bias in an immigratory population. *C. medinalis* is one of the most important migratory pests of rice in Asia^32^. In recent decades, severe population outbreaks have occurred in China, and such outbreaks pose a considerable threat to rice production^33^. Although some studies have carried out long-term monitoring of migratory populations of *C. medinalis*^32,34,35^, systematic analyses of sex ratio bias in immigrating *C. medinalis* have not been conducted, and little is known about the mechanisms underlying sex ratio bias during immigration in *C. medinalis*. *C. medinalis* females do not reach sexually maturity until some time after emergence, whereas males of this species can mate immediately after emergence and engage in multiple mating^36,37^. This observation indicates that female bias in an immigratory population of *C. medinalis* may facilitate the propagation of the species. Since there is no sex difference in phototaxis in *C. medinalis* (see Supplementary Fig. S1), we hypothesize that females emerge earlier than males and that the migratory propensity of female moths is greater than that of male moths in this species, leading to female bias in its immigratory populations.

To test our hypotheses, we used high-altitude searchlight traps to capture *C. medinalis* in the field and determine the numbers of males and females and then investigated emergence time, take-off behaviour, flight capability and energy reserves in the sexes in a laboratory population. The results revealed sex ratio bias in the immigratory population and provided insight into the behavioural and physiological mechanisms. This study illuminates the mechanisms of *C. medinalis* population adaptation and provides valuable information for developing management strategies against this pest.

## Results

### Sex ratio of searchlight-trapped *C. medinalis*

**Figure 1:**
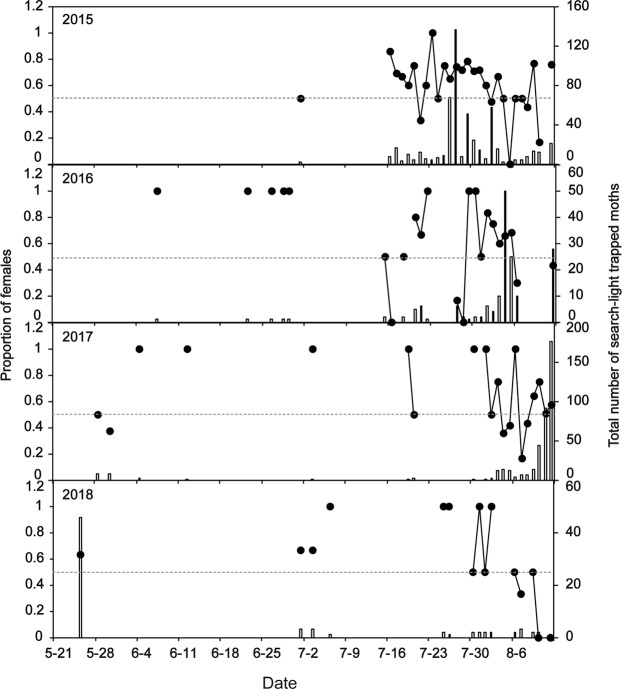
Proportion of females (lines with black circles) and total number of *C. medinalis* (bars) captured by searchlight traps from 2015 to 2018 in Jiangyan. The grey dotted line marks the level representing 50% trapped females.

In the survey conducted in Jiangyan (32°31′44″N, 120°09′5″E), Jiangsu Province, in 2015–2018, 1,170 moths were caught by the search-light trap during the immigration period. Female moths were much more abundant than male moths (females: males = 722:448, Table 1). The ratio of the number of females to the total number of individuals over all days was significantly greater than 0.5 each year (2015: t = 2.676, df = 54, P = 0.012; 2016: t = 2.474, df = 44, P = 0.022; 2017: t = 2.799, df = 38, P = 0.011; 2018: t = 2.218, df = 28, P = 0.044; Fig. 1), indicating a female-biased sex ratio (female:male ratio) in the immigratory population of *C. medinalis* (Table 1).

**Table 1:** Comparison of the numbers of male and female immigrants captured by searchlight traps in Jiangyan from 2015 to 2018. Significant P-values (P < 0.05) are indicated in bold.

Year	Number of females	Number of males	Sex ratio (F:M)	χ^2^	P
2015	343	177	1:0.516	52.992	**<0.001**
2016	100	68	1:0.680	6.095	**0.014**
2017	233	175	1:0.751	8.245	**0.004**
2018	46	28	1:0.608	4.378	**0.036**
Total	722	448	1:0.620	64.168	**<0.001**

### Emergence times of female and male *C. medinalis*

Adults of the laboratory population that were raised from eggs laid on the same day (September 2, 2018) all emerged within a 7-day period (the time from first to last adult emergence). Female moths emerged earlier than male moths. In the first three days of the emergence period, significantly more females than males emerged, whereas on the fourth and fifth days, significantly more males than females emerged (Table 2). Overall, females were slightly more abundant than males (females: males = 1:0.867), but the difference was not significant (Table 2). The emergence times of female and male moths were 2.92 ± 0.07 days and 3.70 ± 0.08 days from the day of first adult emergence, respectively, with females emerging on average 0.78 days earlier than males (t = 7.254, df = 657, P < 0.001).

**Table 2:** Comparison of the numbers of emerging male and female *C. medinalis*. Significant P-values (P < 0.05) are indicated in bold. All of the male and female moths were raised from eggs laid on the same day (September 2, 2018).

Day of emergence (Date)	Number of females	Number of males	Total	Sex ratio (F:M)	χ^2^	P
Day 1 (September 30, 2018)	58	19	77	1:0.328	19.753	**<0.001**
Day 2 (October 1, 2018)	88	52	140	1:0.591	9.257	**0.002**
Day 3 (October 2, 2018)	93	51	144	1:0.548	12.250	**<0.001**
Day 4 (October 3, 2018)	69	97	166	1:1.406	4.723	**0.030**
Day 5 (October 4, 2018)	30	58	88	1:1.933	8.909	**0.003**
Day 6 (October 5, 2018)	14	26	40	1:1.857	3.600	0.058
Day 7 (October 6, 2018)	1	3	4	1:3.000	1.000	0.317
Total	353	306	659	1:0.867	3.352	0.067

### Take-off percentages of female and male *C. medinalis*

Observations of the take-off behaviour of female and male moths of the laboratory population at one to three days after emergence showed that females had a greater emigratory propensity than males. On the first day after emergence, approximately 20% of both female and male moths spiralled vertically to 100 cm above the take-off platform, which was considered a migratory take-off, indicating a high emigratory propensity. However, there was no significant difference in this behaviour between the male and female moths (Table 3). The numbers of female and male moths taking off on the second day after emergence were much larger than those on the first day after emergence. On this day, the migratory take-off percentage of female moths was 74%, significantly higher than that of male moths, 53.33%. This result indicated that female moths have a greater emigratory propensity than male moths on the second day after emergence (Table 3). On the third day after emergence, the migratory take-off percentages of both female and male moths decreased, but the migratory take-off percentage of female moths was significantly higher than that of male moths, indicating a greater propensity of female moths to emigrate (Table 3).

**Table 3:** Take-off percentages of female and male *C. medinalis*. Numbers in parentheses are sample sizes. In each column, * and ns indicate a significant difference and no significant difference, respectively, between females and males at the P < 0.05 level.

Adult age	Female take-off percentage	Male take-off percentage	χ^2^	df	*P*
One day old	20.00 (35)	22.86 (35) ns	0.085	1	0.771
Two days old	74.00 (50)	53.33 (45)*	4.402	1	0.036
Three days old	44.00 (50)	20.00 (55)*	7.000	1	0.008

### Flight capabilities of female and male *C. medinalis*

The flight capability tests of one- to three-day-old male and female moths of the laboratory population, which were performed using flight mills, showed that female moths had a greater flight capability than male moths, indicating that female moths can fly farther than can male moths. The flight duration (t = 0.313, df = 34, P = 0.756) and flight distance (t = 0.477, df = 34, P = 0.636) of two-day-old female moths were slightly greater than those of similarly aged male moths, but the differences were not significant (Table 4). The flight duration (one day old: t = 2.372, df = 53, P = 0.021; three days old: t = 2.178, df = 50, P = 0.038) and flight distance (one day old: t = 2.106, df = 53, P = 0.040; three days old: t = 2.670, df = 50, P = 0.011) of female moths were significantly greater than those of male moths on the first and third days after emergence.

**Table 4:** Flight capability of female and male *C. medinalis*. Data are shown as the mean ± SE. Numbers in parentheses indicate sample sizes. In each column, * and ns indicate a significant difference and no significant difference, respectively, between females and males at the P < 0.05 level.

Adult age	Flight duration (h)		Flight distance (km)	
	Females	Males	Females	Males
1	3.99 ± 0.44 (28)	2.55 ± 0.42 (27)*	5.06 ± 0.65 (28)	3.27 ± 0.54 (27)*
2	3.43 ± 0.68 (24)	3.06 ± 0.91 (12) ns	4.12 ± 0.90 (24)	3.41 ± 1.11 (12) ns
3	6.28 ± 0.88 (22)	3.94 ± 0.66 (30)*	9.94 ± 1.48(22)	5.14 ± 1.02 (30)*

### Lipid and glycogen contents of female and male *C. medinalis*

**Figure 2:**
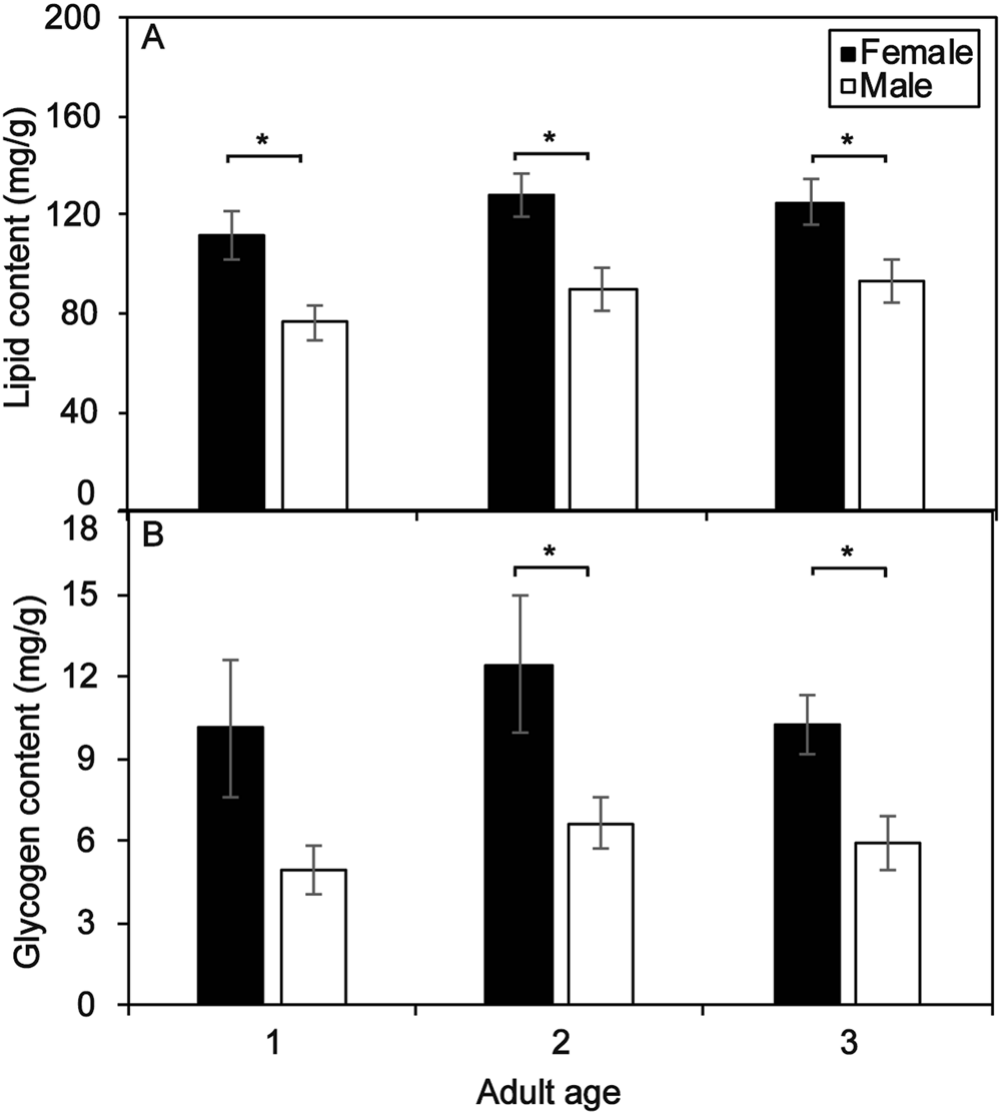
Lipid (**A**) and glycogen (**B**) contents of female and male *C. medinalis*. Data are shown as the mean ± SE. *Indicates a significant difference between females and males at the P < 0.05 level.

The amounts of flight energy substances (lipids and glycogen) of female and male moths of the laboratory population were compared at one to three days after emergence. At that time, the lipid content of female moths was significantly grearter than that of male moths (one day: t = 2.871, df = 18, P = 0.010; two days: t = 3.008, df = 18, P = 0.008; three days: t = 3.666, df = 18, P = 0.002; Fig. 2A). At one day after emergence, the glycogen content of females was greater than that of males, but the difference was not significant (one day: t = 1.936, df = 18, P = 0.069, Fig. 2B). At two and three days after emergence, the glycogen content of females was significantly greater than that of males (two days: t = 3.871, df = 18, P = 0.001; three days: t = 2.986, df = 18, P = 0.008, Fig. 2B). These sex differences in lipid content and glycogen content indicate that female moths store more energy than male moths to maintain long-distance migration, which allows female moths to migrate farther than males.

## Discussion

The survey data from Jiangyan showed that more female than male moths were captured by searchlight traps among the immigrants of *C. medinalis*. Thus, there is female bias in the studied population of *C. medinalis*. We further explored this phenomenon through laboratory tests, and the results supported our hypotheses. We found that compared to the male moths, the female moths emerged earlier, had a greater emigratory propensity and flight capability and had more energy reserves, which led to female bias in the immigratory population of *C. medinalis*.

Female and male reproductive preparation times often vary^38^. As expected, our study found that females of *C. medinalis* emerged earlier than males. Similarly, earlier female emergence has been reported in other insects, such as *Pseudaletia unipuncta*^39^, *O. kirbyi*^40^, and another migratory lepidopteran, *H. armigera*^19^. Moreover, the early emergence of females will inevitably affect dispersal behaviour; that is, the likelihood of leaving the site of origin will be relatively high. In migratory insects, females usually have a greater migration potential and propensity than males^41^. Our study of the take-off behaviour of *C. medinalis* showed that the take-off percentage of young female moths was higher than that of young male moths and that female moths were more likely to take off than male moths, which may indicate more frequent emigration behaviour of female moths than male moths. In addition, because females need to find food and oviposition sites, they usually have strong flight capabilities^42^. Our results indicated that in *C. medinalis*, females have stronger flight capability than males; this pattern is common among other insects, such as *Lygus lineolaris*^43^, *Tetraopes tetrophthalmus*^44^, *Culex pipiens pallens*^45^ and *Mamestra brassicae*^46^. This finding indicates that females have a greater potential for long-distance migratory flight than males. Moreover, considering flight distance, if a male and female begin emigrating at the same time on the same night after emergence, the female may fly farther from the emergence site than the male. Therefore, we believe that compared to males, females emigrate in larger numbers and migrate earlier and farther, which allows them to immigrate to breeding grounds at a higher frequency than males.

Insect behaviour results from complex physiological processes. Insect migration is a process characterized by high energy consumption, with flight muscle development and migratory behaviour requiring extremely large amounts of energy^47,48^. Our findings on the energy reserves of *C. medinalis* showed that the female moths had higher lipid and glycogen reserves than the male moths, which indirectly explained the observed female bias in the immigratory population. Glycogen is an energy substance that insects often use during short-distance flight or take-off ^49^, and in some species, females consume more glycogen than males^31^. In the early stage of the emergence period, the glycogen content of female moths was significantly greater than that of male moths in *C. medinalis*, which may explain why female moths exhibited take-off more often than male moths. Lipids are the main fuel for long-term insect migration^50,51^. Females of *C. medinalis* have more lipid reserves than males to provide adequate fuel for long-distance migration. The energy reserves of *C. medinalis* females are conducive to migration and ensure that the females can migrate over long distances, which may explain why more females than males were captured by the searchlight traps during immigration.

Sex differences in migration strategies may affect the population structure of migratory populations^52^. In general, females are responsible for most of the gene flow and dispersal of a population^41^. From an evolutionary perspective, large numbers of immigratory females are beneficial in terms of population growth for several reasons. First, the stronger flight capability of female moths than of male moths can increase the range of population dispersal, which can reduce female intraspecific competition by allowing oviposition at very distant locations^53^. Second, different immigration times of female moths and male moths can reduce inbreeding and increase opportunities to mate with distantly related males, thereby reducing the risk of inbreeding depression. Third, because male moths can mate with many female moths^36,37^, larger numbers of immigrating female moths than immigrating male moths will not affect female mating success but will promote female mating with males with a strong flight capability, which could result in superior offspring and improve the adaptability of the population during evolution. In terms of population development, the physiological and behavioural responses of *C. medinalis* have led to the evolution of females that migrate earlier than males, which may represent an adaptive ecological strategy used by insects to increase fertility and prevent inbreeding depression.

In summary, our study explored female bias in an immigratory population of *C. medinalis*. We obtained observational and experimental evidence that female moths migrate earlier than male moths, that the migratory propensity of female moths is greater than that of male moths, and that the energy reserves of female moths are conducive to long-distance migration, all of which result in significant female bias in the immigratory population.

However, the reasons for the sex difference in the arrival time of *C. medinalis* are complex, and our understanding of female bias during immigration in *C. medinalis* is far from complete. In the future, it will be necessary to study sex differences in migratory pests by focusing on physiological and molecular regulatory mechanisms to better understand sex ratio bias. Such efforts will enhance our understanding of the migration strategies of pests and be valuable for formulating prevention and treatment recommendations in order to facilitate the sustainable control of pest populations.

## Methods

### Sex ratio investigation of searchlight-trapped moths

Survey data were obtained for *C. medinalis* in Jiangyan, Jiangsu Province, China (32°31′44″N, 120°09′5″E), using vertically pointing searchlight trap to attract and capture high-altitude migrants (up to 500 m above ground level)^54^. The data were collected from May to September each year from 2015 to 2018. The searchlight trap automatically turned on at 19:00 (Beijing Time, BJT) and off at 07:00 (BJT) the next day. The numbers of male and female adults of *C. medinalis* were recorded every day, and the ratio of the number of females to the total number of individuals was calculated. Since *C. medinalis* does not overwinter in Jiangyan, the moths captured by the searchlight trap in the early stage were mainly from the immigrant population. Moreover, it takes approximately one month for *C. medinalis* to emerge from the egg and reach the adult stage^55,56^. According to the field survey data of the moth, there were almost no *C. medinalis* moths in early July or before (Supplementary Fig. S2), indicating that there was almost no local adult emergence in early August. Because our focus was on the immigratory population, we analysed the sex ratio bias of only the immigrants that were captured by searchlight traps from May 21 to August 12 according to moth abundance (see Supplementary Fig. S2) and ovary development (taking 2017 and 2018 as examples, see Supplementary Table S1).

### Survey of male and female emergence times

Larvae of *C. medinalis* had been collected previously from rice paddy fields in Nanjing, Jiangsu Province, China, and reared using wheat seedlings^55^. Pupae were removed from the seedlings and transferred to a transparent plastic box (16 cm in length, 24 cm in width and 22 cm in height), the bottom of which was filled with moist cotton wool to maintain a high relative humidity (RH). Pairs of newly emerged male and female adults were transferred to 500 ml transparent cups containing absorbent cotton wool soaked in a 5% honey solution, which was provided as a supplemental food. The cups were covered with plastic film, and the adults oviposited on the film. All insects were reared in RXZ intelligent artificial climate chambers (Ningbo Jiangnan Instrument Factory, China) at 26 ± 0.5 °C and an 80–90% relative humidity (RH) with a photoperiod of 14 L:10 D^57^. Eggs laid on the same day (September 2, 2018) were collected and raised under the same conditions described above. Upon adult emergence, the numbers of female and male adults were recorded daily, and the time from the egg to adult emergence was considered the duration of the immature stage of *C. medinalis*. We studied a total of 659 pupae.

### Observations of take-off behaviour in females and males

Pupae were collected from rice paddy fields in Nanning, Guangxi Autonomous Region, China and transferred into a transparent plastic box (16 cm in length, 24 cm in width and 22 cm in height), the bottom of which was filled with moist cotton wool to maintain a high relative humidity. The insects were reared in the laboratory. After emergence, females and males were separately housed in 500 ml transparent cups containing absorbent cotton wool soaked in a 10% honey solution, which was provided as a supplemental food at a density of 5 moths per cup. One- to three-day-old female and male moths were used to determine the take-off percentage. Before their take-off behaviour was observed, the moths were placed in a climate chamber (Ningbo Jiangnan Instrument Factory, China) at 26 ± 1 °C and a 70–80% RH for one hour. The take-off behaviour of *C. medinalis* from dusk to night may include take-off for migration and take-off for dispersal (foraging, courtship, etc.). According to previous field observations, flight behaviour with a more than 100 cm spiral, vertical rise above the take-off platform is an effective take-off behaviour for migration. Here, the number of adults that completed an effective migratory take-off was recorded, and the take-off percentage was calculated. During the take-off observations, the light source was composed of 20 rows of fluorescent lamps (36 V/40 W) and 2 incandescent lamps (12 V/40 W). Since radar observations have shown that *C. medinalis* generally takes off after 19:00^35^, all take-off observation periods began at 19:00 (BJT). To ensure the consistency of the lighting conditions across observations, the light intensity was changed by gradually extinguishing 20 parallel fluorescent lamps (2 lamps every 3 min) and connecting the incandescent lamps to a potentiometer to create artificial evening light simulating natural conditions, and the indoor light intensity was gradually decreased from 1,000 lx to 0.1 lx over 45 min. The light intensity changes during the observation period were measured with a TES-1330A illuminometer. In all observations, the light source was located 200 cm above the take-off platform to eliminate the effect of heat from the light source on the internal temperature of the take-off platform. Five females or five males were placed on the take-off platform for each observation event, and a total of 135 females and 130 males were observed.

### Measurement of the flight capability of females and males

Larvae of *C. medinalis* had previously been collected from rice fields in Nanjing, China, and reared using wheat seedlings^56^. Pupae were reared using the methods detailed above. After emergence, each female was paired with a male, and the pairs were transferred to 500-ml transparent cups containing absorbent cotton wool soaked in 10% honey solution, which was provided as a supplemental food. Flight tests of adults were conducted with a 24-channel computer-interfaced flight mill system (Jiaduo Science, Industry and Trade Co., Ltd., Hebi, Hebei, China) that could automatically record total flight distance and duration. Adult moths of 1–3 days of age were used for the tethered-flight tests. A total of 74 female moths and 69 male moths were tested. Each adult was tethered following a procedure described in a previous study^57^. The experimental moths were mildly anaesthetized with ether before attachment to a tether arm. The lamellar scales around the junction between the metathorax and abdomen were brushed off using a soft brush pen. Then, a hollow plastic tether with a diameter of 0.80 cm and a length of 1.5 cm was attached to the dorsal metathorax using Pattex superglue (Henkel Adhesive Co. Ltd., China). The flight capability of the adult moths was not affected by this treatment and was similar to that of other adult moths that were neither anaesthetized nor glued to a tether^58^. The flight direction of each tethered moth remained perpendicular to the arm of the round-about flight mill. The light conditions were the same as those established for the take-off observations. The lights were turned off at 20:00 and then turned on at 6:00. The flight environment was maintained at 26 ± 1 °C and an 80–90% RH, and the flight tests were carried out from 19:00 to 7:00 (BJT).

### Determination of the lipid and glycogen contents of females and males

**Figure 3:**
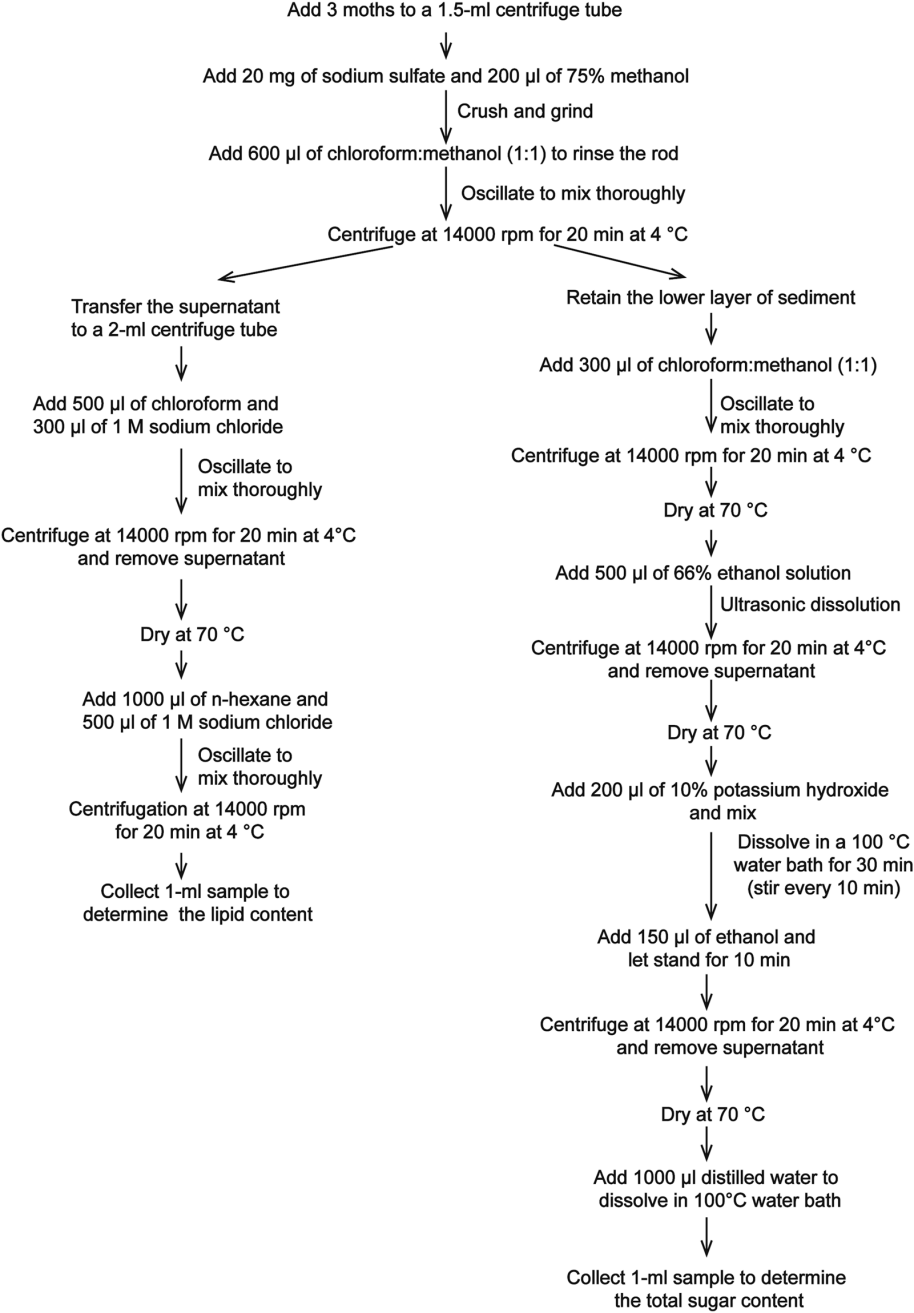
The methods used for extraction and separation of lipids and glycogen^59^.

Female and male moths of 1–3 days of age were used to determine lipid and glycogen contents. Ninety moths of each sex were used. The lipid and glycogen contents of one-, two- and three-day-old female and male moths were determined. The procedure for extracting and separating the lipid and glycogen was based on work by Lorenz^59^ (Fig. 3).

Lipid content was measured using the sulfophosphovanillin method^60^. Samples were added to 10 ml centrifuge tube. Each tube was then supplemented with 1,000 μl of n-hexane and 1 ml of sulfuric acid and then placed in a boiling water bath for 10 min. After cooling the tubes to room temperature, 5 ml of phosphoricillin solution was added to each sample. Samples were measured against a cholesterol standard at 530 nm using a microplate reader (SpectraMax M5, Molecular Devices, LLC, United States).

Glycogen content was measured using the anthrone method described by Zhao, *et al*.^61^. Briefly, 1 ml of separated sample was placed in a 10 ml centrifuge tube and then cooled in an ice bath; 5 ml of 1 mg/ml anthrone reagent was then added to each tube. Then, the tubes were placed in boiling water for 10 min, removed and cooled in an ice bath. The samples were measured against a glucose standard at 620 nm using a microplate reader (SpectraMax M5, Molecular Devices, LLC).

### Data analysis

All data are presented as means ± standard errors (SEs). The ratio of females to males captured by the searchlight trap was compared to a 1:1 ratio using a Chi-squared test, and the ratio of the number of females to the total number over all days was compared to a value of 0.5 using a t-test. The ratios of females to males at different emergence times were compared to a 1:1 ratio using a Chi-squared test. The take-off percentages of male and female adults were analysed with a Chi-squared test. The flight capability and lipid and glycogen contents of male and female adults were analysed with t-tests. All statistical analyses were performed with IBM SPSS Statistics (V21) software.

## Supplementary information

Supplementary Fig. S1, Fig. S2 and Table S1

## Data Availability

The datasets analysed in the current study are available from the corresponding authors upon reasonable request.
