# Supplementary material for: Female bias in an immigratory population of Cnaphalocrocis medinalis moths based on field surveys and laboratory tests
Source: Sci Rep. 2019 Dec 5;9:18388. doi: 10.1038/s41598-019-54721-x (PMC6895038; doi:10.1038/s41598-019-54721-x)
Supplement: Supplementary file 1 — Supplementary Fig. S1, Fig. S2 and Table S1 [file 41598_2019_54721_MOESM1_ESM.pdf]

Female bias in an immigratory population of *Cnaphalocrocis medinalis* moths based on field surveys and laboratory tests

Jia-Wen Guo [1], Fan Yang [1,2], Ping Li [1], Xiang-Dong Liu [1], Qiu-Lin Wu [1,3], Gao Hu [1,*], Bao-Ping Zhai [1,*]

1. Key Laboratory of Integrated Management of Crop Diseases and Pests (Ministry of Education), College of Plant Protection, Nanjing Agricultural University, Nanjing, China

2. Vegetable Research Institute, Wuhan Academy of Agricultural Science and Technology, Wuhan, China.

3. State Key Laboratory for Biology of Plant Diseases and Insect Pests, Institute of Plant Protection, Chinese Academy of Agricultural Sciences, Beijing, China

* Corresponding authors: Gao Hu, Bao-Ping Zhai

Street address: No. 1 Weigang Road, Xuanwu District, Nanjing 210095, China

E-mail address: bpzhai@njau.edu.cn (B. Zhai), hugao@njau.edu.cn (G. Hu)

Telephone number: +86(0)2584396204 (B. Zhai), +86(0)2584396381 (G. Hu)

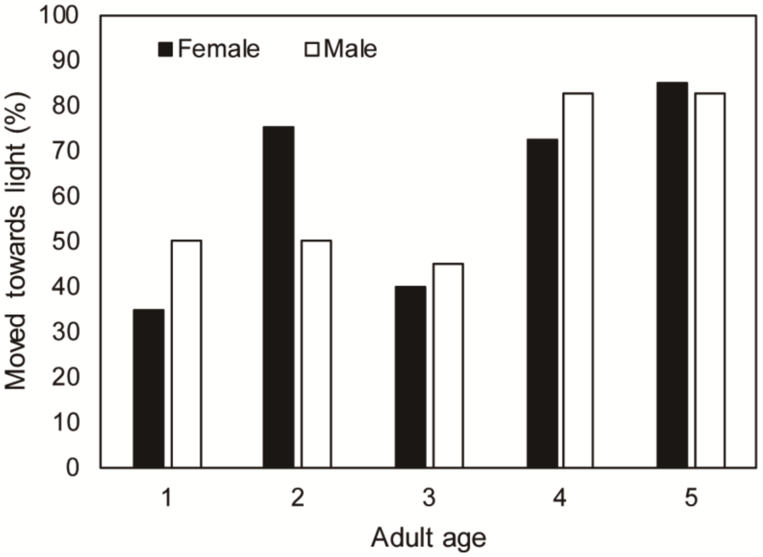

Supplementary Fig. S1. The percentage of adult *C. medinalis* moths that first moved towards the light. A total of 280 healthy moths were selected and tested. The equipment used to study phototaxis was similar to a positive phototaxis experimental apparatus used in a previous experiment, with slight modifications [1]. Newly emerged unpaired female and male moths were transferred daily to an experimental cup on the left side of an opaque test channel (containing a cotton ball soaked with a 5% honey solution). The light source consisted of a trap light (PS-15II type, Jiaduo Science, Industry and Trade Co., Ltd., Hebi, Henan, China) commonly used for pest monitoring and control (15 W; lλ=320-680 nm). The lights were turned on for 12 hours (from 19:00 to 7:00) each day. Once the light was turned off, the number of adults moving towards the right observation cup in each treatment was recorded. The experiment was carried out at 26 ± 1 °C. One- to five-day-old *C. medinalis* showed no sex differences in phototaxis (1-day-old: χ^2=0.921, df=1, P=0.337; 2-day-old: χ^2=2.667, df=1, P=0.102; 3-day-old: χ^2=0.102, df=1, P=0.749; 4-day-old: χ^2=1.147, df=1, P=0.284; 5-day-old: χ^2=0.092, df=1, P=0.762).

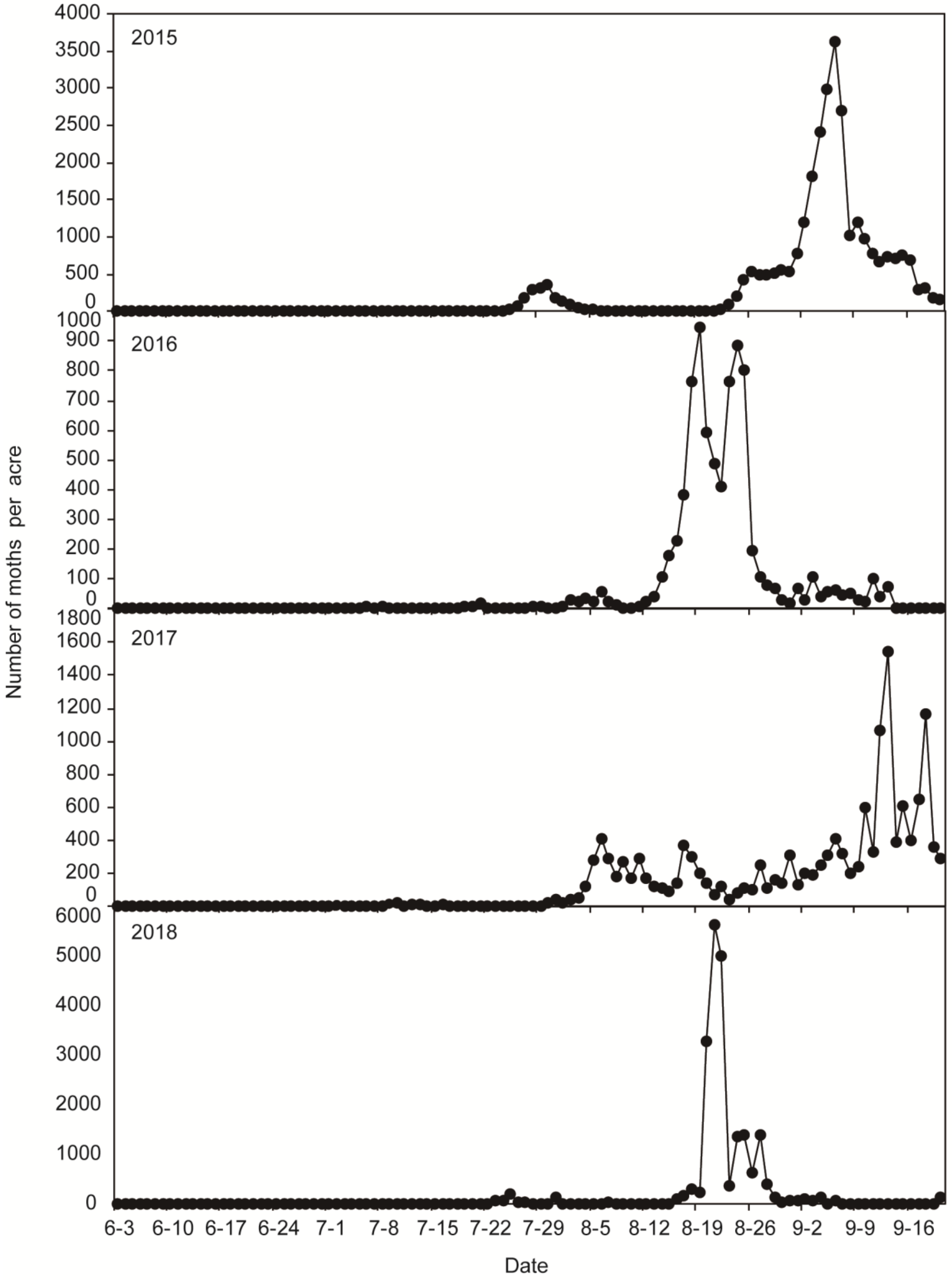

Supplementary Fig. S2. Population dynamics of *C. medinalis* moths in Jiangyan, China. The abundance of moths was recorded by using a survey carried out at 6 am (BJT), with a method based on that described by Wang *et al.* [2].

Supplementary Table S1. Abundance and female ovarian development of *C. medinalis* moths in Jiangyan, China.

Year	Period	No. of moths per acre	No. of moths dissected	Population size of moths in each stage of ovarian development			Population characteristics
				Level I	Level II	≥ Level III	
2017	27 July 15 Aug	1150	91	3.30	13.19	83.52	Mostly immigrants
2018	27 July 12 Aug	51	26	0	19.23	80.77	Mostly immigrants

Female moths of *C. medinalis* were collected from rice fields and dissected once every two days to determine their ovarian developmental stages according to the criteria described by Zhang *et al*. [3]. Before August 12, the *C. medinalis* moths in the field in Jiangyan, China, were the immigratory population.

References

1. Wan, G. J. *et al.* Reduced geomagnetic field may affect positive phototaxis and flight capacity of a migratory rice planthopper. *Anim. Behav.* **121**, 107-116, doi:10.1016/j.anbehav.2016.08.024 (2016).
2. Wang, F. Y. *et al.* Determining the migration duration of rice leaf folder (*Cnaphalocrocis medinalis* (Guenée)) moths using a trajectory analytical approach. *Sci. Rep.* **7**, 39853, doi:10.1038/srep39853 (2017).
3. Zhang, X. X., Lu, Z. Q. & Geng, J. G. Application of ovarian dissection of female *Cnaphalocrocis medinalis* moths in prediction and forecasting system. *Entomological Knowledge* **16**, 97-99 (1979).
